# Changes in and correlates of cannabis-involved substance use treatment admissions age 50 and older, 2000–2021

**DOI:** 10.3389/fpubh.2025.1592551

**Published:** 2025-07-03

**Authors:** Namkee G. Choi, C. Nathan Marti, Bryan Y. Choi

**Affiliations:** ^1^Steve Hicks School of Social Work, University of Texas at Austin, Austin, TX, United States; ^2^Department of Emergency Medicine, Philadelphia College of Osteopathic Medicine and Bayhealth, Dover, DE, United States

**Keywords:** older adults, cannabis use disorder, cannabis use treatment, cannabis legalization, substance use treatment

## Abstract

**Background and aims:**

Cannabis use among U.S. older adults has risen rapidly over the past two decades. This study examined the changes in and correlates of cannabis-involved substance use treatment admissions among this demographic.

**Methods:**

Using the 2000–2021 concatenated Treatment Episode Data Set-Admissions (TEDS-A) age 50+ (*N*=5,593,004), we fitted joinpoint regression models to examine changes in the percent of cannabis-involved admissions of all substance use admissions. We used multinomial and binary logistic regression models to examine the demographic and treatment-related correlates of cannabis-primary admissions and cannabis-secondary/tertiary admissions.

**Results:**

During the study period, the number of cannabis-involved admissions increased substantially, while their share of all admissions increased and then decreased as other drug-related admissions increased. The annual percentage changes (APC) show that the shares of cannabis-involved admissions of all admissions between 2000 and 2012 increased for the 50–64 age group and then decreased between 2012 and 2021. In the 65+ age group, the shares increased between 2000 and 2016 (APC=5.2) and then plateaued. Compared to no-cannabis admissions, the likelihood (relative risk ratio) of all cannabis-involved admissions was higher among males, black people, residents of states where medical or recreational cannabis use was legal, and referrals from healthcare providers and court/criminal legal systems. The likelihood (adjusted odds ratio [AOR]) of cannabis-primary admissions was higher among those age 65+ (aOR=1.04, 95%CI=1.00–1.08), black people (aOR=1.34, 95% CI=1.32–1.36), Hispanic people (aOR=1.26, 95% CI=1.23–1.29), residents of states with medical cannabis laws, and those who were referred by healthcare providers and legal systems.

**Implications:**

Cannabis-involved admissions are projected to continue to increase as cannabis use continues to increase. More effective regulations and enforcement of delta-9-tetrahydrocannabinol potency and research on cannabis harms and poly-substance use are needed to protect the health of older adults who turn to cannabis for its purported health benefits. Increased availability and accessibility of treatment infrastructure are also needed.

## Introduction

As of March 10, 2025, medicinal cannabis is legal in 39 U.S. States, the District of Columbia, and three territories, and personal (recreational) cannabis use is legal in 24 states, the District of Columbia, and three territories (1). Cannabis is decriminalized (no arrest or criminal record for the first-time possession of a small amount of cannabis for personal consumption) in two states where cannabis remains illegal (1).

With cannabis use legalized or decriminalized in most states, epidemiologic data from the National Surveys on Drug Use and Health (NSDUH) showed increasing use rates in the adult population, from 10.4% in 2002 to 15.3% in 2017 and 23.0% in 2022 (2, 3). Cannabis use frequency, specifically daily/near-daily use, also increased during these years, from 1.9% in 2002 to 4.2% in 2017 to 5.8% in 2022 (2, 3). The number of people reporting 21 + days of cannabis use in the past month surpassed the number of people reporting the same frequency of alcohol use in 2022 (4). Cannabis use disorders (CUD) per *DSM-5* criteria (5) among cannabis users range from 20 to 30%, depending on the study sample, with higher rates among more frequent users (2, 6, 7). A systematic review and meta-analysis also found that among individuals using medicinal cannabis in the past 6–12 months, the prevalence of CUD was 29% (95% CI = 21–38%) (8).

Although individuals age 18–49 comprise the majority of cannabis users, those in the 50 + age group showed the most increase in use rates, i.e., from 4.3 and 1.1% in 2013 to 8.9 and 3.4% in 2019, respectively, in the 50–64 and 65 + age groups (9). Our analysis of the 2022 NSDUH showed that 18.0 and 8.0% of individuals in the 50–64 and 65 + age groups, respectively, reported past-year cannabis use. This rapid increase in use rate is not surprising given that those age 50 + are more likely to turn to cannabis for therapeutic benefits for health problems, such as chronic pain, insomnia, and chemotherapy-induced nausea and vomiting, which tend to be more common among older than younger adults. Studies have shown that around 60% of all medical cannabis registrants in different states are age 50 and older (10, 11). The booming cannabis industry has also targeted older adults to advertise cannabis’s health and wellness benefits (12).

Contrary to claims of health benefits, a scoping review of older adults found inconsistent therapeutic effects of medical cannabis use, with harmful associations outnumbering beneficial ones and greater frequencies of depression, anxiety, cognitive impairment, substance use and problematic substance use, accidents/injuries, and acute healthcare use among cannabis users than nonusers (13). Another review also found that available evidence regarding the therapeutic effects of medical cannabis use among older adults is inconsistent and tends to rely on self-report and uncontrolled studies (14). With legalization, more potent cannabis products (i.e., higher concentrations of Δ^9^-tetrahydrocannabinol or THC) have become readily available, contributing to adverse health outcomes such as intoxication, cannabis hyperemesis syndrome, disrupted cognitive functioning, and worsening mental health problems; in particular, depression, anxiety, and psychosis (15–17). Older adults may be especially vulnerable to these and other harmful effects on cardiovascular, gastrointestinal, and pulmonary functions (18, 19). Cannabis can also lead to cannabis-drug interactions (e.g., cannabis-warfarin) and impaired driving and related injuries in older adults (20–23). Since older medical users, compared to nonmedical users, also tend to use cannabis more frequently, they may be subjected to a higher risk of CUD and associated health problems, and were found to have higher rates of healthcare visits and discussing their drug use with a healthcare professional than non-medical users (24).

The overall share of any substance use treatment among adults with CUD tends to be low (<13.0% in 2005–2013 [25] and 16.5% in 2022 [2]). Wu et al. (25) found that among individuals age 50 + years with lifetime CUD, only one out of five received any substance use treatment, with less than one-half of them for CUD specifically, though treatment receipts were higher for those with other co-occurring substance use disorders and physical and mental health problems, and lower for Asian-Americans, women, and college-educated adults. However, more recent data showed increased treatment admissions among older adults. For example, a study based on Treatment Episode Data Set-Admissions (TEDS-A: https://www.samhsa.gov/data/data-we-collect/teds-treatment-episode-data-set), compiled by the Substance Abuse and Mental Health Services Administration [SAMHSA], found that between 2000 and 2017, the treatment cases age 55 + increased by 203.7% as compared to 13.0% among younger-adult cases (26). Further, older adults showed greater increases relative to younger adults in proportions admitted for cannabis and cocaine/crack use and a relative decrease in admission for alcohol and opiates (26). A study of TEDS-A admissions age 55 + between 2012 and 2017 also found that the absolute number of cannabis-involved treatment admissions increased over the study period, although the yearly proportion of cannabis-involved admissions (14.1% on average) of all admissions age 55 + did not change (27).

Contrary to these increases among older adults, studies show that CUD treatment utilization has declined in recent years, particularly among adolescents and young adults, while cannabis use has increased (28–31). Mennis et al. (31–33) found that after legalization, compared to before, the positive association between low-risk perception and cannabis use was strengthened, whereas the positive association between treatment admissions and cannabis use was suppressed. They also found that the decrease in admissions among young adults stemmed from a significant reduction in the proportion of young-adult criminal justice referrals, likely due to falling cannabis-related arrests following recreational cannabis legalization. However, adult admissions in the 2000–2017 TEDS-A did not show any significant relationships between admissions and recreational cannabis legalization (34). Moreover, Bass et al. (30), in their study of all publicly funded substance use treatments delivered in California from 2010 to 2021, found an increase in the probability of admission to CUD treatment for those referred from the criminal justice system as well as for black and Hispanic individuals, despite the overall decrease in admissions. Another study based on 2007–2019 TEDS-A also found significant rate increases in criminal justice system referrals for black and Hispanic/Latino adults at 6 years after policy change and black juveniles at 2 to 6 years after policy change in legalized states (35).

While these studies are informative, further research is necessary on individuals in the 50 + age group who enter CUD treatment, as they have shown the greatest increase in cannabis use rates. In the present study, we used the 2000–2021 concatenated TEDS-A (36) to first examine changes in the shares (i.e., percentages) of all cannabis-involved admissions and cannabis-primary admissions among all treatment admissions age 50 + and then in three age groups (50–54, 55–64, and 65+) over the 22 years. Second, we examined the demographic and treatment-related correlates of cannabis-primary admissions and cannabis-secondary/tertiary admissions compared to the admissions that did not involve cannabis. Third, we examined the demographic and treatment-related correlates of cannabis-primary admissions versus cannabis-secondary/tertiary admissions. We posited the following hypotheses: (H1) the shares of all cannabis-involved and cannabis-primary admissions age 50+, those age 65 + in particular, would have steadily increased between 2000 and 2021; (H2) the likelihood of both cannabis-primary and cannabis-secondary/tertiary admissions, compared to the admissions not involving cannabis, would be higher among males, black people, and those referred to by healthcare providers and the court/criminal justice systems; and (H3) among all cannabis-involved admissions, the likelihood of cannabis-primary admissions would be higher among those age 65+, those without prior treatment histories, and in medical cannabis legal (MCL) and personal/recreational cannabis legal (RCL) states. The findings of this study will contribute to the knowledge base of CUD-involved substance use treatment admissions among late middle-aged and older adults.

## Materials and methods

### Data source

TEDS-A includes treatment admissions of individuals age 12 + to facilities that are licensed or certified by a state substance use agency and receive state alcohol and/or drug agency funds (including federal block grant funds to provide care for people with a substance use disorder) or to facilities that are administratively tracked for other reasons (36). A few states have not participated in TEDS-A every year. For example, Oregon has not provided data since 2015, and Washington has not provided data since 2018.

Each TEDS record represents a treatment episode (e.g., an individual admitted to treatment twice within a calendar year is counted as two admissions) and provides demographic, clinical, and substance use characteristics of the person admitted to treatment services. Between 2000 and 2021, a total of 40,559,305 admissions age 12 + were recorded, with a steady increase from 2000 (*N* = 1,748,957) to 2008 (*N* = 2,064,820), followed by a steady decrease from 2008 to 2015 (*N* = 1,694,055), an increase from 2015 to 2018 (*N* = 2,031,116), a slight decline in 2019 (*N* = 1,889,755), and then a notable decrease during the COVID pandemic years of 2020 (*N* = 1,545,201) and 2021 (*N* = 1,482,543). However, the shares of admissions age 50 + of all admissions increased steadily from 7.7% in 2000 to 20.8% in 2021, averaging 13.8% (*N* = 5,593,004; 7.1% [*N* = 2,865,283] age 50–54 years; 5.8% [*N* = 2,362,875] ages 55–64 years; and 0.9% [*N* = 364,846] ages 65 + years) over 22 years. Analysis of de-identified, publicly available TEDS-A data was exempt from review by the authors’ institutional review boards.

### Measures

#### Cannabis cases

TEDS-A lists up to three problem substances—primary, secondary, and tertiary--that led to the treatment episode. In this study, we divided all admissions age 50 and older into three groups: those not including cannabis as the primary, secondary, or tertiary substance (referred to as no cannabis admissions); those where cannabis was listed as the primary problem substance (cannabis-primary admissions); and those where cannabis was listed as the secondary or tertiary problem substance (cannabis-secondary/tertiary admissions). All cannabis-involved admissions refer to the admissions where cannabis was listed as the primary, secondary, or tertiary problem substance.

#### Sociodemographic factors

These included: (1) age group (50–54, 55–64, and 65+); (2) gender; (3) race/ethnicity (non-Hispanic white, non-Hispanic black, Hispanic, American Indian/Alaska Native, Asian or Pacific Islander, and other people of multi or unknown race); (4) education (<high school, high school diploma or GED, some college, bachelor’s degree); (5) employment status (descriptive purpose only); (6) living arrangement (homelessness [no fixed address or living at a shelter], supervised living setting, and independent living [living alone or with others without supervision]); and (7) resident state’s cannabis law (cannabis illegal, MCL only, and RCL).

#### Treatment-related characteristics

These were: (1) number of previous drug and/or alcohol treatment episodes (0, 1, 2, 3+, missing); (2) treatment referral sources (self or other individual; alcohol/drug abuse care provider); other healthcare provider (physician, psychiatrist, or other licensed healthcare professional, health/mental healthcare service settings); employer/employee assistance program (EAP)/educational agency/other community entity (including social service and religious organizations and self-help groups); and legal entity (court/criminal justice referral/driving under the influence); (3) treatment setting at admission (grouped into three: detoxification, residential rehabilitation, and ambulatory/outpatient treatment); and (4) other most frequently-involved substances reported at admission (alcohol, cocaine/crack, heroin, other opiates and synthetics, and methamphetamine/speed; no, yes for each).

### Analysis

Analyses were conducted with Stata 18/MP (StataCorp, College Station, TX) and Joinpoint Regression Program, version 5.2.0 (Calverton, MD). First, we described percentages of all cannabis-involved admissions and then cannabis-primary admissions of all admissions in three age groups (50–54, 55–64, and 65+) and by admission year. For brevity in the table presentation, we provided the average over 2 years, i.e., 2000/2001 through 2020/2021. To test H1 (examination of any significant trends in cannabis-involved admissions from 2000 through 2021), we fitted joinpoint models for annual percentage changes (APC) in three age groups, first for all cannabis-involved admissions and then for cannabis-primary admissions. Second, we used χ^2^ tests to compare sociodemographic and treatment-related characteristics and other substance involvement by three cannabis groups (no cannabis, cannabis primary, and cannabis secondary/tertiary). Third, to test H2 (examination of the correlates of cannabis-primary and cannabis-secondary/tertiary admissions vs. no-cannabis admissions), we fitted a multivariable, multinomial logistic regression model. Fourth, to test H3 (correlates of cannabis-primary vs. cannabis-secondary/tertiary admissions), we fitted a binary logistic regression model. For a sensitivity analysis, we also fit a binary logistic regression model for the 65 + age group to examine whether or not correlates of cannabis-primary admissions differ in this age group.

As a preliminary analysis, we used the variance inflation factor (VIF) from linear regression models to assess multicollinearity among covariates (37). VIF diagnostics indicated that multicollinearity was not a concern (VIF ≤ 2.5) for all variables except the admission year variable, which had VIFs ranging from 2.06 to 4.91. Given the fact that the admission year was a categorical variable with 11 categories, we applied a higher VIF cut-off of 5. Our sensitivity analyses of both multinomial and binary logistic regression models, excluding the admission year variable, revealed no significant deviation from the models that included the variable. Multinomial logistic regression results are presented as relative risk ratios (RRR) with 95% CI, and binary logistic regression results are presented as adjusted odds ratios (aOR). Statistical significance was set at *p* < 0.05.

## Results

### Cannabis-involved treatment admissions ages 50 and older, 2000–2021

Table 1 shows that cannabis-involved admissions averaged 14.8% of all admissions age 50 + (16.9, 13.3, and 7.5% of the admissions in the 50–54, 55–64, and 65 + age groups, respectively) over the 22-year period. The data also indicate that cannabis-involved admissions steadily increased from 9.6% in 2000/2002 to 16.9% in 2012/2013, followed by a decline to 14.2% in 2020/2021. However, cannabis-involved admissions age 65 + steadily increased from 4.0% in 2000/2001 to 9.5% in 2018/2019 before decreasing to 8.6% in 2020/2021. The drop in 2020 across all three age groups corresponds to the start of the COVID-19 pandemic, but there was an uptick in 2021 for all three age groups. The data show that 20% (20.0% for the 50–54 age group, 19.7% for the 55–64 age group, and 23.4% for the 65 + age group) of all cannabis-involved admissions were cannabis-primary admissions. Additional analysis also showed that 34.0% of the cannabis-primary admissions were cannabis-only admissions (i.e., no other substances involved), indicating that 7.8% of all cannabis-involved admissions (and 1.2% of all admissions age 50+) were for cannabis-only admissions.

**Table 1:** Share of cannabis and the other five most frequently involved substances among substance use treatment admissions age 50+, 2000–2021 (% of all admissions age 50 + in the years shown).

	Total, 2000–2021 5,593,004 (100%)	2000–2001 275,454 (4.92%)	2002–2003 324,615 (5.80%)	2004–2005 358,254 (6.41%)	2006–2007 430,412 (7.70%)	2008–2009 509,403 (9.11%)	2010–2011 528,160 (9.44)	2012–2013 547,741 (9.79%)	2014–2015 561,395 (10.04%)	2016–2017 669,865 (11.98%)	2018–2019 765,208 (13.68%)	2020–2021 622,497 (11.13%)
Any cannabis (primary, secondary, or tertiary)	14.76	9.57	10.75	12.16	13.66	14.76	16.09	16.85	16.67	16.14	15.62	14.15
50–54 years	16.85	11.99	12.97	14.32	15.72	16.89	18.37	19.14	18.84	18.35	17.70	16.31
55–64 years	13.34	7.0	8.33	9.81	11.46	12.66	13.95	15.0	15.20	14.92	14.83	13.45
65 + years	7.45	4.0	4.35	5.0	5.85	5.89	7.07	7.93	8.38	8.92	9.45	8.64
Cannabis primary	2.96	1.85	2.11	2.43	2.72	3.09	3.48	3.58	3.45	3.29	3.01	2.42
50–54 years	3.39	2.28	2.47	2.81	3.09	3.50	3.91	4.09	3.90	3.78	3.44	2.89
55–64 years	2.63	1.34	1.68	2.00	2.34	2.64	3.04	3.13	3.12	2.97	2.80	2.18
65 + years	1.74	1.14	1.29	1.18	1.23	1.74	1.95	1.95	1.93	2.03	2.05	1.71
Alcohol	66.58	76.97	74.56	72.70	71.46	72.31	73.11	72.07	67.36	61.96	42.85	48.29
Cocaine/crack	23.96	18.90	21.89	24.89	27.38	26.42	25.68	24.57	23.01	23.12	24.70	21.25
Heroin	20.68	19.15	20.18	19.77	19.49	18.28	17.15	18.06	22.61	24.68	25.41	18.36
Other opiates and synthetics	6.90	3.04	4.12	5.11	5.82	6.72	8.09	8.39	7.90	8.09	7.58	6.60
Methamphetamine/ speed	5.45	1.67	2.45	2.98	3.78	3.63	3.90	4.98	6.10	7.28	8.24	8.46

Total column percentages add up to more than 100, as many cases involved more than one substance.

Table 1 also shows a steady decrease in the shares of alcohol-involved admissions from 77.0% in 2000/2001 to 62.0% in 2016/2017, dropping to 42.9% in 2018–2019 and 48.3% in 2020/2021. In contrast, there was a steady increase in the shares of admissions involving cocaine/crack, heroin, other opiates/synthetics, and especially methamphetamine/speed (from 1.7% in 2000/2021 to 8.5% in 2020/2021). Overall, among all admissions age 50 and older, alcohol was the most common substance involved, followed by cocaine/crack, heroin, and cannabis. However, cannabis-involved admissions increased faster than those involving cocaine/crack and heroin.

**Figure 1:**
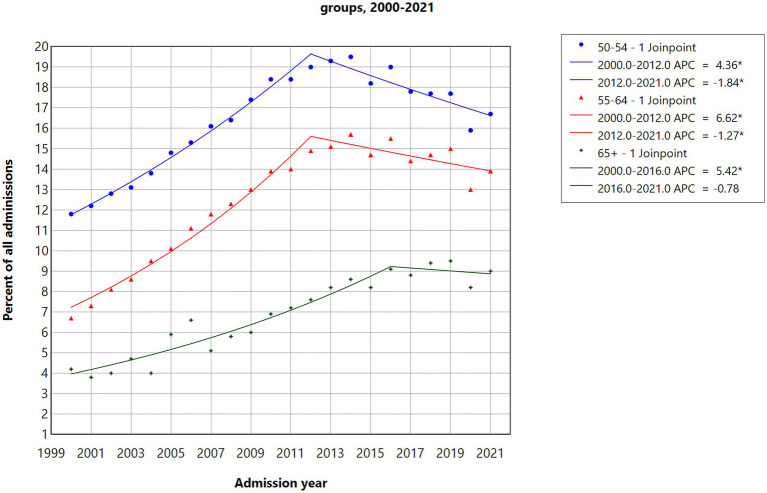
Joinpoint regression models for admissions with cannabis as the primary, secondary, or tertiary substance in three age groups, 2000–2021.

Joinpoint modeling results in Figure 1 illustrate significant (*p* < 0.05) increases in the shares of cannabis-involved admissions for the 50–54 (APC = 4.36) and 55–64 (APC = 6.62) age groups from 2000 to 2012, followed by significant decreases from 2012 to 2021 (APC = −1.84 for the 50–54 age group and APC = -1.27 for the 55–64 age group). For the 65 + age group, significant increases (APC = 5.42) were observed from 2000 to 2016, followed by a nonsignificant decline from 2016 to 2021. These results partially support H1, as the shares of cannabis-involved admissions stopped increasing after 2012 for the 50–64 age group and after 2016 for the 65 + age group.

**Figure 2:**
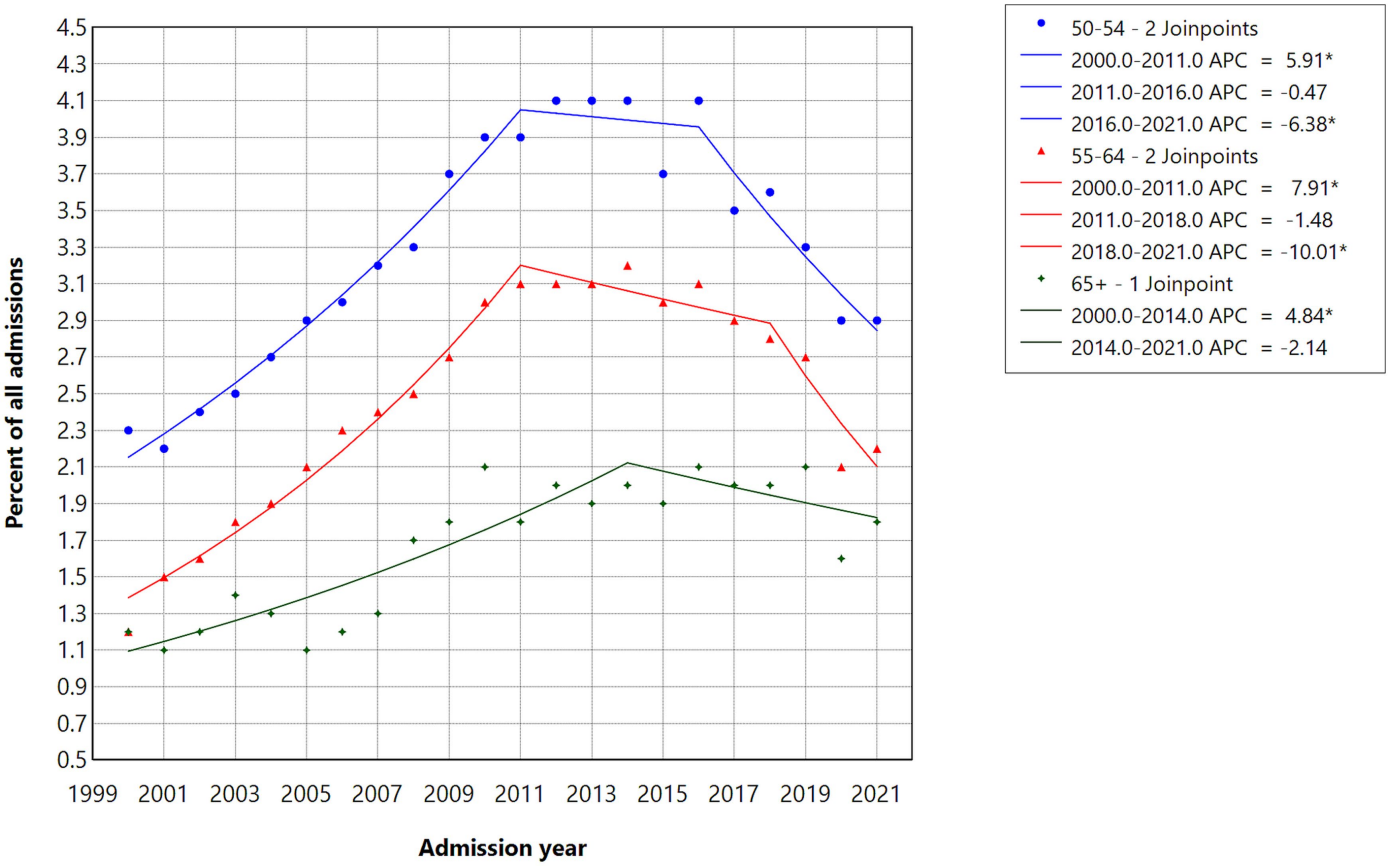
Joinpoint regression models for admissions with cannabis as the primary substance in three age groups, 2000–2021.

Figure 2 illustrates significant increases in the shares of cannabis-primary admissions for the 50–54 (APC = 5.91) and 55–64 (APC = 7.91) age groups from 2000 and 2011, followed by nonsignificant decreases from 2011 to 2016 for the 50–54 age group and from 2011 to 2018 for the 55–64 age group. The reductions were significant for the 50–54 age group from 2016 to 2021 (APC = −6.38) and for the 55–64 age group from 2018 to 2021 (APC = −10.01). The increase was significant for the 65 + age group from 2000 to 2014 (APC = 4.84), followed by a nonsignificant decrease from 2014 to 2021.

### Sociodemographic and treatment-related characteristics of cannabis involvement

Table 2 shows that compared to no-cannabis admissions, cannabis-involved admissions included higher proportions of those age 50–54, males, black people, and ambulatory/outpatients but lower proportions of those age 65+, non-Hispanic white people, and Hispanic people. Cannabis-primary admissions included higher proportions of those working and independently living. Compared to no-cannabis admissions or cannabis-secondary/tertiary admissions, cannabis-primary admissions also had a smaller proportion of those who were homeless and higher proportions of those who lived in the states where cannabis was illegal, had no previous treatment admissions, and were court/criminal justice system referrals. Further analysis showed that about 35% of the cannabis-primary admissions that were court/criminal justice system referrals were probation/parole cases.

**Table 2:** Characteristics of admissions age 50+, 2000–2021, by cannabis involvement state.

	All	No cannabis	Cannabis primary	Cannabis secondary or tertiary
	5,593,004 (100%)	4,767,600 (85.24%)	165,616 (2.96%)	659,788 (11.80%)
Age group (years; %)				
50–54	51.23	49.97	58.71	58.46
55–64	42.25	42.95	37.46	38.38
65+	6.52	7.08	3.83	3.16
Gender (%)				
Female	26.44	27.02	25.56	22.46
Male	73.51	72.92	74.41	77.52
Missing	0.06	0.06	0.04	0.03
Race/ethnicity (%)				
Non-Hispanic white	52.68	53.17	50.96	49.59
Black	28.00	26.96	33.08	34.28
Hispanic	11.19	11.53	8.65	9.39
American Indian/Alaska Native	2.11	2.11	1.74	2.22
Asian or Pacific Islander	0.57	0.58	0.61	0.49
Other or more than two races	5.44	5.65	4.96	4.03
Education (%)				
Less than high school	25.16	24.70	28.63	27.66
GED or high school diploma	41.04	40.73	43.06	42.82
Some college	18.53	18.31	18.50	20.09
Bachelor’s degree	9.01	9.44	5.72	6.66
Missing	6.26	6.82	4.10	2.77
Employment status (%)				
Working full- or part-time	18.31	18.15	24.42	17.92
Unemployed	28.10	27.77	28.35	30.43
Not in the labor force	47.06	46.99	43.14	48.49
Missing	6.53	7.09	4.09	3.16
Living arrangement (%)				
Homeless	17.95	18.03	10.69	19.16
Dependent living	11.36	11.05	12.86	13.25
Independent living	60.39	59.93	68.98	61.53
Missing	10.30	10.99	7.47	6.05
Resident state’s cannabis law (%)				
Cannabis use illegal	10.37	10.14	14.68	10.92
Only medical use legal	26.10	25.78	33.03	26.68
Recreational use legal	63.53	64.08	52.30	62.40
Previous treatment episode (%)				
None	29.33	29.19	45.77	26.27
Once	17.60	17.34	21.58	18.47
Twice	10.93	10.78	10.22	12.21
3 + times	27.06	27.09	14.34	30.04
Missing	15.07	15.60	8.09	13.01
Referral source (%)				
Individual (including self-referral)	43.08	44.27	25.21	38.96
Alcohol/drug counselor	10.74	10.89	5.25	11.03
Healthcare provider	9.21	9.21	8.87	9.29
Employer/school/community resources	10.19	9.83	15.23	11.47
Court/criminal justice system	21.62	20.20	42.35	26.74
Missing	5.16	5.59	3.09	2.52
Treatment setting (%)				
Detoxification	29.06	31.33	4.58	18.82
Residential rehabilitation	16.13	15.80	9.94	20.08
Ambulatory/outpatient care	54.80	52.87	85.48	61.10

Omnibus *χ*^2^ tests showed that the differences among the three groups were significant at *p* < 0.001 for all variables.

### Other types of substances

Table 3 shows the distribution of the six most frequently involved substances among no-cannabis, cannabis-primary, and cannabis-secondary/tertiary admissions. Alcohol was by far the most commonly involved substance in all three groups of admissions; however, alcohol was present in a significantly lower proportion of cannabis-primary admissions than the other two groups of admissions (38.9% vs. 66.5% of no-cannabis admissions and 74.2% of cannabis-secondary/tertiary admissions). Cocaine/crack was the second most commonly involved substance, with cannabis-secondary/tertiary admissions showing the highest percentage (39.5%). Heroin was the third most frequently involved substance, with no-cannabis admissions showing the highest proportion (22.2%) and cannabis-primary admissions the lowest proportion (3.4%). Other opiates/synthetics were present in smaller proportions of cannabis-primary and cannabis-secondary/tertiary admissions compared to no-cannabis admissions. Conversely, methamphetamines/speed were present in larger proportions of cannabis-primary and cannabis-secondary/tertiary admissions compared to no-cannabis admissions.

**Table 3:** Involvement of other substances by cannabis involvement status.

	All	No cannabis	Cannabis primary	Cannabis secondary or tertiary
	5,593,004 (100%)	4,767,600 (85.24%)	165,616 (2.96%)	659,788 (11.80%)
Alcohol	66.58	66.49	38.92	74.19
Cocaine/crack	23.96	21.95	20.06	39.46
Heroin	20.68	22.17	3.39	14.24
Other opiates and synthetics	6.90	7.19	3.18	5.67
Methamphetamine/speed	5.45	4.67	9.91	9.90

Omnibus *χ^2^* tests showed that the differences among the three groups were significant at *p* < 0.001 for all variables.

### Correlates of cannabis-involved admissions: results from multinomial logistic regression

The second and third columns of Table 4 show that the likelihood of both cannabis-primary and cannabis-secondary/tertiary admissions was 1.75 times higher in 2018/2019 than in 2000/2001, although the likelihood of cannabis-primary admissions was slightly down in 2020/2021. Compared to no-cannabis admissions, the likelihood of both cannabis-primary and cannabis-secondary/tertiary admissions was higher among males (RRR = 1.35, 95%CI = 1.33–1.36 for cannabis-primary; RRR = 1.32, 95%CI = 1.31–1.33 for cannabis-secondary/tertiary); black people (RRR = 1.50, 95%CI = 1.48–1.52 for cannabis-primary; RRR = 1.10, 95%CI = 1.09–1.11 for cannabis-secondary/tertiary); and American Indians/Alaska Natives (RRR = 1.19, 95%CI = 1.14–1.24 for cannabis-primary; RRR = 1.25, 95%CI = 1.23–1.27 for cannabis-secondary/tertiary). The likelihood of both cannabis-primary and cannabis-secondary/tertiary admissions was also higher among MCL or RCL state residents (MCL RRR = 1.10, 95%CI = 1.09–1.12; RCL RRR = 1.12, 95%CI = 1.11–1.14 for cannabis-primary; MCL RRR = 1.04, 95%CI = 1.03–1.05; RCL RRR = 1.08, 95%CI = 1.06–1.09 for cannabis-secondary/tertiary). As for the referral sources, the likelihood was higher for those who were referred by healthcare providers (RRR = 1.37, 95%CI = 1.34–1.39 for cannabis-primary; RRR = 1.08, 95%CI = 1.07–1.09 for cannabis-secondary/tertiary), employers/school/community resources (RRR = 1.95, 95%CI = 1.91–1.98 for cannabis-primary; RRR = 1.12, 95%CI = 1.11–1.13 for cannabis-secondary/tertiary), and court/criminal legal systems (RRR = 1.86, 95%CI = 1.84–1.89 for cannabis-primary; RRR = 1.13, 95%CI = 1.13–1.14 for cannabis-secondary/tertiary). However, the likelihood of referrals from substance use counselors was lower (RRR = 0.94, 95%CI = 0.92–0.97 for cannabis-primary; RRR = 0.95, 95%CI = 0.95–0.96 for cannabis-secondary/tertiary). These results largely support H2.

**Table 4:** Correlates of cannabis involved admission cases: multinominal and binary logistic regression results.

	Cannabis primary RRR (95% CI)	Cannabis secondary or tertiary RRR (95% CI)	Cannabis primary aOR (95% CI)
	Vs. No cannabis		Vs. Cannabis secondary/tertiary
Admission year: vs. 2000–2001			
2002–2003	1.16 (1.11–1.20)***	1.11 (1.09–1.13)***	0.98 (0.93–1.03)
2004–2005	1.30 (1.25–1.35)***	1.18 (1.16–1.20)***	0.97 (0.93–1.01)
2006–2007	1.36 (1.31–1.40)***	1.27 (1.24–1.29)***	0.97 (0.93–1.02)
2008–2009	1.55 (1.50–1.61)***	1.38 (1.36–1.40)***	1.03 (0.98–1.07)
2010–2011	1.87 (1.81–1.93)***	1.53 (1.50–1.55)***	1.11 (1.06–1.15)***
2012–2013	2.02 (1.95–2.08)***	1.66 (1.63–1.68)***	1.09 (1.05–1.14)***
2014–2015	2.01 (1.94–2.08)***	1.73 (1.70–1.76)***	1.08 (1.04–1.13)***
2016–2017	1.94 (1.88–2.00)***	1.75 (1.73–1.78)***	1.05 (1.01–1.09)*
2018–2019	1.74 (1.68–1.79)***	1.76 (1.73–1.79)***	0.94 (0.91–0.98)**
2020–2021	1.42 (1.37–1.47)***	1.75 (1.72–1.78)***	0.84 (0.81–0.88)***
Age group: vs. 50–54 years			
55–64	0.74 (0.73–0.74)***	0.79 (0.79–0.80)***	0.94 (0.93–0.95)***
65+	0.41 (0.40–0.42)***	0.45 (0.44–0.45)***	1.04 (1.01–1.08)*
Gender: vs. Female			
Male	1.35 (1.33–1.36)***	1.32 (1.31–1.33)***	0.99 (0.97–1.00)
Missing	1.12 (0.86–1.46)	1.05 (0.90–1.23)	1.31 (0.95–1.82)
Race/ethnicity: vs. Non-Hispanic white			
Black	1.50 (1.48–1.52)***	1.10 (1.09–1.11)***	1.34 (1.32–1.36)***
Hispanic	0.99 (0.97–1.01)	0.87 (0.86–0.88)***	1.26 (1.23–1.29)***
American Indian/Alaska Native	1.19 (1.14–1.24)***	1.25 (1.23–1.27)***	0.99 (0.95–1.04)
Asian or Pacific Islander	0.77 (0.72–0.82)***	0.80 (0.77–0.83)***	1.09 (1.00–1.18)
Other or multiple races	0.96 (0.94–0.98)**	0.93 (0.91–0.94)***	1.20 (1.17–1.24)***
Education: vs. < high school			
GED or high school diploma	0.87 (0.85–0.88)***	0.94 (0.93–0.95)***	0.92 (0.91–0.94)***
Some college	0.86 (0.85–0.88)***	0.98 (0.97–0.99)***	0.88 (0.86–0.89)***
Bachelor’s degree	0.54 (0.53–0.56)***	0.70 (0.69–0.71)***	0.77 (0.74–0.79)***
Missing	0.28 (0.27–0.29)***	0.54 (0.53–0.55)***	1.13 (1.09–1.17)***
Living arrangement: vs. homelessness			
Dependent living	0.99 (0.97–1.01)	0.98 (0.97–0.99)**	1.06 (1.03–1.08)***
Independent living	1.07 (1.05–1.09)***	0.98 (0.98–0.99)***	1.16 (1.13–1.18)***
Missing	0.71 (0.69–0.73)***	0.81 (0.80–0.82)***	1.23 (1.19–1.27)***
Resident state’s cannabis law: vs. Illegal			
Only medical use legal	1.10 (1.09–1.12)***	1.04 (1.03–1.05)***	1.08 (1.05–1.10)***
Recreational use legal	1.12 (1.11–1.14)***	1.08 (1.06–1.09)***	0.91 (0.89–0.92)***
Previous treatment episode: vs. None			
Once	0.97 (0.96–0.98)***	1.14 (1.13–1.14)***	0.83 (0.82–0.85)***
Twice	0.88 (0.85–0.88)***	1.19 (1.18–1.20)***	0.73 (0.72–0.75)***
3 + times	0.70 (0.69–0.71)***	1.21 (1.20–1.22)***	0.59 (0.58–0.60)***
Missing	0.71 (0.69–0.73)***	1.26 (1.25–1.27)***	0.85 (0.83–0.87)***
Referral source: vs. Individual			
Alcohol/drug counselor	0.94 (0.92–0.97)***	0.95 (0.95–0.96)***	0.98 (0.96–1.01)
Healthcare provider	1.37 (1.34–1.39)***	1.08 (1.07–1.09)***	1.29 (1.26–1.33)***
Employer/school/community resources	1.95 (1.91–1.98)***	1.12 (1.11–1.13)***	1.64 (1.60–1.67)***
Court/criminal justice system	1.86 (1.84–1.89)***	1.13 (1.13–1.14)***	1.55 (1.53–1.58)***
Missing	0.45 (0.43–0.46)***	0.73 (0.71–0.74)***	1.49 (1.43–1.55)***
Treatment setting: vs. Detoxification			
Residential rehabilitation	3.29 (3.20–3.38)***	1.87 (1.85–1.89)***	1.82 (1.77–1.88)***
Ambulatory/outpatient care	6.97 (6.80–7.14)***	2.15 (2.14–2.17)***	3.64 (3.54–3.74)***
**Alcohol involvement**	0.12 (0.11–0.12)***	1.49 (1.48–1.50)***	0.09 (0.09–0.09)***
**Cocaine/crack involvement**	0.37 (0.37–0.38)***	2.22 (2.21–2.24)***	0.22 (0.22–0.23)***
**Heroin involvement**	0.04 (0.03–0.04)***	0.60 (0.59–0.60)***	0.06 (0.06–0.06)***
**Other opiates and synthetics involvement**	0.14 (0.13–0.14)***	0.95 (0.94–0.96)***	0.17 (0.17–0.18)
**Methamphetamine/speed involvement**	0.47 (0.46–0.48)***	2.40 (2.37–2.42)***	0.21 (0.20–0.21)***
Model statistics	*N* = 5,593,004; Likelihood ratio *χ*^2^(88) = 603041.76, *p* < 0.001		*N* = 825,404; Likelihood ratio *χ*^2^(44) = 225,776.77, *p* < 0.001

*p < 0.05; **p < 0.01; ***p < 0.001.

The likelihood of both cannabis-primary and cannabis-secondary/tertiary admissions was lower among the 55–64 and 65 + age groups than in the 50–54 age group, Asian/Pacific Islanders and other/multi-race cases, and those with high school or higher education. Both cannabis-primary and cannabis-secondary/tertiary admissions, compared to no-cannabis admissions, had a higher likelihood of receiving rehabilitation and ambulatory/outpatient services than detoxification. The likelihood of cannabis-primary admissions was lower, but the likelihood of cannabis-secondary/tertiary admissions was higher among those with any previous treatment history. The likelihood of cannabis-primary admissions was lower among those with other substance involvement, but the likelihood of cannabis-secondary/tertiary admissions was higher among those with alcohol, cocaine/crack, and methamphetamine/speed involvement.

### Correlates of cannabis-primary versus cannabis-secondary/tertiary admissions: results from binary logistic regression

The fourth column of Table 4 shows that compared to cannabis-secondary/tertiary admissions, the odds of cannabis-primary admissions were higher in 2010/2011 through 2016/2017 but lower in 2018/2019 and 2020/2021. The odds of cannabis-primary admissions were higher among those age 65 + (aOR = 1.04, 95%CI = 1.00–1.08), black people (aOR = 1.34, 95% CI = 1.32–1.36), Hispanic people (aOR = 1.26, 95% CI = 1.23–1.29), those who were not homeless, those who were referred by healthcare providers, employers/school/community resources, and court/criminal legal systems, MCL state residents (aOR = 1.08, 95% CI = 1.05–1.10), and those in rehabilitation or ambulatory care settings. The odds of cannabis-primary admissions were lower among those with high school or higher education, those with any previous treatment history, RCL state residents, and those with other substance involvement. These results partially support H3. Logistic regression results among admissions age 65 and older did not deviate much from the above results in terms of black people, education, referral sources, previous treatment history, service settings, RCL state residency, and other substance involvement. However, in the 65 + age group, the odds of cannabis-primary admissions were lower starting in 2014/2015 and among males, and MCL state residency was not a significant factor.

## Discussion

Between 2000 and 2019, the number of substance use treatment admission cases age 50 + increased steadily. During this period, although alcohol remained the most frequently involved substance, its share of all admissions declined substantially, while the shares of admissions involving the five next most frequently reported substances—cocaine/crack, heroin, cannabis, other opiates/synthetics, and methamphetamine/speed (in descending order of share)—increased. In 2020–2021, the total number of admissions decreased substantially, likely reflecting service disruptions associated with the COVID-19 pandemic, rather than a decline in cannabis use or related problems, as studies indicated that self-reported cannabis use increased during the pandemic (38). Over 22 years, the number of cannabis-involved admissions grew steadily, even though their share of all admissions began to decline from 2014/2015. Nonetheless, cannabis-involved admissions grew at a faster rate than those involving cocaine/crack or heroin, accounting for approximately one in seven admissions among older adults. Admissions involving other opiates/synthetics and methamphetamine/speed increased at even faster rates than cannabis-involved admissions, which aligns with the sharp rise in overdose deaths involving opioids and psychostimulants between 2012 and 2019 (39). Nevertheless, each of these two categories of substances continued to represent fewer than one in ten admissions in this age group.

Consistent with the previous study finding (27), one-fifth of cannabis-involved admissions were cannabis-primary cases. Of all cannabis-involved admissions, 92% involved other substances. Alcohol was the most involved, but cocaine/crack was also involved in more than 20% of cannabis-primary admissions and nearly 40% of cannabis-secondary/tertiary admissions, heroin in 3% of cannabis-primary admissions and 14% of cannabis-secondary/tertiary admissions, and methamphetamines/speed in nearly 10% of both cannabis-primary and cannabis-secondary/tertiary admissions. The high rates of cocaine/crack and heroin involvement in cannabis-secondary/tertiary admissions suggest that these substances were likely the primary substances.

Although the number of cannabis-involved treatment admissions age 50 + continued to rise over the past two decades, the share of these admissions relative to all treatment admissions did not show a corresponding increase and varied by age group. Notably, among those aged 50–64, the share of cannabis-involved admissions declined significantly between 2012 and 2021. This suggests that while absolute numbers continued to grow, the rate of increase was outpaced by admissions involving other substances, particularly cocaine/crack, heroin, other opiates/synthetics, and psychostimulants. The decline or plateau in the share of cannabis-involved admissions among older adults may also reflect broader societal shifts, including cannabis legalization, decriminalization, and declining risk perceptions. The year 2012, which marked the beginning of the downward trend in cannabis-involved admission shares for the 50–64 age group, coincides with the legalization of recreational cannabis use in Colorado and Washington. Since then, many additional states have enacted MCL and RCL and implemented decriminalization measures.

The declining share of cannabis-involved treatment admissions may also reflect a reduced prevalence of moderate or severe CUD. The NSDUH data show that the prevalence of moderate or severe CUD among adults (age 18+) decreased from 6.7% in 2002 to 3.1% in 2022, although the prevalence of mild CUD increased from 1.9% in 2017 to 3.9% in 2022 (2, 3). However, older adults represent one of the fastest-growing groups using medical cannabis and may be at heightened risk for developing CUD, particularly in the presence of co-occurring medical and psychiatric conditions (14, 18). A longitudinal study of veterans receiving care through the Veterans Health Administration between 2005 and 2019 found that the enactment of MCL and RCL was associated with increased CUD prevalence in states that adopted these policies, especially among individuals with chronic pain (40, 41). Older adults who use cannabis to manage chronic pain or other chronic health conditions may therefore be especially vulnerable to cannabis-related harms, including the development or exacerbation of CUD (19), prompting the need for treatment.

Regarding the correlates of cannabis-involved admissions, this study found a higher likelihood of admission among individuals aged 50 and older residing in MCL or RCL states. Older cannabis users in these states may have used cannabis more extensively and frequently, contributing to an elevated risk of adverse outcomes that necessitated treatment. Additionally, MCL and RCL states may offer more accessible or better-resourced treatment systems, as the availability and accessibility of substance use treatment services for older adults vary considerably across states (42). As expected, admissions age 50 + were more likely to be referred by healthcare providers, reflecting more frequent contacts with healthcare providers in this age group. Another noteworthy finding is the relatively high likelihood of referrals from the court or criminal justice system, particularly for cannabis-primary admissions, compared to self-referrals. This is consistent with previous findings from California’s TEDS-A, which showed an increase in the probability of treatment entry for CUD through criminal justice referrals following the implementation of RCL (30). Further research is warranted to explore the personal and policy contexts surrounding court or criminal justice system referrals for cannabis-involved admissions, especially in cases where cannabis is the primary substance.

Differences between cannabis-primary and cannabis-secondary/tertiary admissions are also noteworthy. As hypothesized, admissions age 65 + compared to those age 50–54, those without prior treatment episodes, and those in MCL states were more likely to be cannabis-primary rather than cannabis-secondary/tertiary. In general, the odds of cannabis-primary admissions versus cannabis-secondary/tertiary admissions were also higher between 2010/2011 and 2016/2017 but were lower in 2018/2019 and 2020/2021. However, the odds of cannabis-primary admissions age 65 + were lower starting in 2014/2015. These patterns likely reflect not only changes in cannabis use but also increases in the use of other substances in this population. For example, a TED-A-based study reported that between 2012 and 2019, the numbers of both heroin-only and heroin-cocaine admissions age 55 + increased 2.3-fold, heroin-methamphetamine admissions increased seven-fold, and first-time heroin-methamphetamine admissions rose 18-fold (43). The lower odds of cannabis-primary, relative to secondary/tertiary, admissions in RCL states further suggest that, in these states, cannabis was less often the primary substance of concern at treatment entry, possibly due to the rising prevalence of other drug use. Another important difference between cannabis-primary and cannabis-secondary/tertiary admissions is the treatment settings. Cannabis-primary cases were significantly more likely to receive ambulatory care, while cannabis-secondary/tertiary cases were significantly more likely to be at residential treatment settings. This suggests that cannabis-primary cases need less supervision and probably more capacity to manage symptoms independently, while cannabis-secondary/tertiary cases need more intensive intervention.

Multivariable analysis results consistently showed a higher likelihood of cannabis-involved admissions among black older adults (compared to non-Hispanic white older adults) and those with lower education. Of all cannabis-involved admissions, black older adults and those with lower education had higher odds of cannabis-primary than cannabis-secondary/tertiary admissions. Over the past decade, research has shown high-frequency cannabis use and CUD prevalence to be higher among black and Native American adults and those with lower education (44–47). However, a study also found that RCL enactment increased the odds of CUD among non-Hispanic white people, Hispanic people, and people of other races/ethnicities but not among black people (48). The greater prevalence of cannabis use and CUD among black adults may be linked to social motives and financial stress, both of which are associated with perceived barriers to cannabis cessation (49, 50). The higher treatment admission rates for black people could have been due to persistent racial disparities in arrest rates. A study of cannabis arrest rates from 2009 through 2016 in Colorado and Washington found that despite a general decline in cannabis arrests for nearly all racial groups following the legalization, substantial racial disparities persisted, especially in Colorado (51). To be specific, the ratio of black-to-white arrest rates for cannabis possession was 24.9 to 1 in 2009 and dropped to 4.2 to 1 in 2016 (51). Another study that examined cannabis possession arrest ratios from January 2000 through December 2019 in 43 U. S. States also found that although overall arrest rates declined following legalization and decriminalization policies, racial disparities in arrest rates remained (52). Additionally, the overall high proportion of admissions involving individuals experiencing homelessness is a concern that warrants further investigation, particularly given the intersection of housing instability, substance use, and access to care.

The study’s limitations are due to the following constraints: First, TEDS-A includes only admissions to publicly supported treatment programs; thus, admissions to private treatment programs that are likely to serve those with more financial or other resources are excluded. Second, because of the lack of data on the patterns or the numbers of clinics reporting data over time, we could only report the changes in the shares/percentages, not rates, of cannabis-involved admissions. Third, since TEDS-A reports on admissions, not individuals, and does not include information on transfer from one program to another (e.g., from detoxification to residential rehabilitation or outpatient treatment), the extent of duplication of cases is unknown. Fourth, TEDS-A has large amounts of missing data for many variables (e.g., use frequency, psychiatric problems) that prevent a more meaningful analysis of the contributions of these variables. Fifth, TEDS-A does not provide data on cannabis consumption methods (e.g., smoking, vaping, edibles, dabbing) despite the rapidly growing array of cannabis products, limiting more detailed analysis.

## Conclusion

Over the past two decades, we observed a consistent increase in the number of cannabis-involved treatment admissions among adults aged 50 and older. However, the proportion of cannabis-involved admissions relative to all substance use treatment admissions declined over the past decade, likely due to rising admissions for heroin, other opioids, and psychostimulants. The number of cannabis-involved admissions is expected to continue rising, as CUD prevalence grows in parallel with the expanding number of states adopting MCL or RCL and increasing access to commercial cannabis outlets. Despite limited evidence supporting cannabis’s therapeutic benefits, older adults with chronic health conditions, especially those with low income or limited access to healthcare, may be drawn to cannabis use in an effort to address unmet health needs. As use increases, voluntary cannabis use may escalate into compulsive behaviors, with individuals developing CUD despite awareness of its harmful consequences and struggling to reduce or stop use (53). The high prevalence of poly-substance use, homelessness, and court/criminal justice referrals among cannabis-secondary/tertiary admissions underscores the complex needs of older adults who co-use cannabis with other illicit substances. Stronger regulation and enforcement of THC potency, along with expanded research on the harms of cannabis and poly-substance use, are urgently needed to protect public health, particularly that of older adults who turn to cannabis for perceived health benefits. Additionally, increased investment in publicly funded treatment infrastructure is essential to support individuals lacking access to other care resources.

We found consistently increased numbers of cannabis-involved treatment admissions among the 50 + age group over the past two decades, although the shares/percentages of cannabis-involved admissions of all admissions decreased over the past decade, likely due to increased admissions involving heroin and other opiates and psychostimulants. The number of cannabis-involved admissions is likely to continue to rise, as CUD prevalence is projected to grow as the number of states with MCL or RCL and access to legal and commercial cannabis outlets increases. Despite limited evidence on cannabis’s therapeutic effects, older adults with chronic health problems, especially low-income people and those with limited access to healthcare systems, may be more easily drawn to using cannabis, trying to meet their unmet healthcare needs. With increasing use, voluntary cannabis-seeking behaviors can become compulsive habits, and those with CUD may be aware of the harmful consequences of use but find it difficult to exert restraint and cease use (53). The high prevalence of poly-substance use, homelessness, and criminal justice involvement, especially among cannabis-secondary/tertiary admissions, signals complex, unmet needs among older adults who co-use cannabis with other illicit substances. To better protect public health, especially the health of older adults who turn to cannabis for perceived therapeutic benefits, several targeted strategies are warranted. First, stronger regulation and enforcement of THC potency limits are essential in products marketed as medicinal. Second, expanded research and dissemination of scientifically rigorous data on the potential harms of cannabis and poly-substance use are urgently needed. Third, tailored outreach efforts through senior centers, public housing, home health agencies, homeless shelters, and telehealth platforms to identify and engage socially isolated older adults at risk for CUD. Fourth, geriatric addiction training for primary care providers, mental health clinicians, and substance use counselors to enhance recognition and management of CUD and poly-substance use in older populations. Fifth, publicly funded investments must prioritize age-appropriate, integrated treatment infrastructure, including behavioral therapies tailored for older adults with comorbid chronic conditions.

## Data Availability

The data analysed in this study is available from: Treatment Episode Data Set-Admissions (TEDS-A; Substance Abuse and Mental Health Services Administration [SAMHSA]: https://www.samhsa.gov/data/data-we-collect/teds-treatment-episode-data-set). Further inquiries can be directed to the corresponding author.
